# Investigating the association between gut microbiota and venous thromboembolism mediated by immune cells: A mediation Mendelian randomization study

**DOI:** 10.1097/MD.0000000000047709

**Published:** 2026-02-20

**Authors:** Chao Wang, Xiaofa Wu, Jia Wang, Bojian Fei, Hui Jiang

**Affiliations:** a Department of Gastrointestinal Surgery, Affiliated Hospital of Jiangnan University, Wuxi, Jiangsu, China; b Wuxi School of Medicine, Jiangnan University, Wuxi, Jiangsu, China.

**Keywords:** gut microbiota, immune cells, Mendelian randomization, single-nucleotide polymorphisms, venous thromboembolism

## Abstract

Recent studies have indicated a correlation between gut microbiota (GM) and venous thromboembolism (VTE). However, the specific effects of GM on VTE-related immune factors remain largely unknown. Thus, we conducted a Mendelian randomization (MR) analysis to explore the relationships between GM, immune cells, and VTE. This mediation MR study utilized the summary-level data of respective genome-wide association study for 207 taxa and 205 bacterial pathways, 731 immune cell traits, and VTE. This MR design was deployed, and comprehensive sensitive analyses were followed to validate the robustness of the results. We used the inverse-variance weighted, Bayesian-weighted MR, MR-Egger regression, MR-Egger intercept test, Cochrane *Q* test, and leave-one-out analysis as the primary methods. We identified a suggestive association between 16 bacterial traits and VTE risk, with 3 taxa affecting VTE through 2 types of immune cells as mediators. For example, “c_Bacteroidia” was implicated in reducing the risk of VTE via “DN (CD4^−^ CD8^−^) NKT % lymphocyte” cells. This study suggested the role of the specific GMs on the VTE risk, emphasizing the potential mediatory function of GM-related immune cells.

## 1. Introduction

Venous thromboembolism (VTE) affects around 10 million people globally each year, with incidence increasing sharply with age.^[1]^ VTE is primarily caused by major surgeries and active cancer, which together account for nearly 50% of cases.^[2]^ The pathogenesis of VTE is rooted in the Virchow triad: stasis of blood flow, hypercoagulability, and endothelial damage.^[3]^ Research indicates that alterations in the gut microbiota (GM) are closely associated with thrombus formation. Understanding and modifying the GM-related risk factors for VTE is crucial to prevent thrombotic events and their long-term consequences.

The human GM has emerged as a crucial host partner in recent years, often regarded as a “new organ” influencing various bodily systems, including the brain, lungs, and cardiovascular system, mainly through its metabolites.^[4]^ Research showed that probiotics can significantly modulate immune and anti-inflammatory mechanisms. Beneficial bacteria within the gut enhance immune responses by promoting the activation of immune cells. Conversely, dysbiosis, characterized by reduced microbial diversity, can trigger inflammation by altering T helper cell differentiation and increase the risk of disease.^[5]^ The GM also plays a key role in immunometabolism by producing metabolites such as short-chain fatty acids, bile acids, and trimethylamine N-oxide.^[6]^ Additionally, it can generate from dietary sources such as phosphatidylcholine, L-carnitine, and choline, which are abundant in Western diets.^[7]^ Elevated trimethylamine N-oxide levels have been linked to the increased risk of thrombotic events.^[8]^ Emerging evidence further highlights the complex interplay between coagulation and inflammation, where the coagulation cascade activates the immune system, promoting thrombosis through a process known as immunothrombosis. The GM potentially serves as a reservoir for systemic inflammation and coagulation activation by facilitating the translocation of lipopolysaccharide from gram-negative bacteria into the bloodstream.^[9]^ While growing evidence highlights the GM’s role in chronic diseases,^[10]^ its specific connections with VTE remain poorly understood. Disruption of the GM ecosystem may lead to alterations in immune responses in the human body, potentially triggering the onset of VTE. Therefore, identifying the association between the host immune microenvironment and the gut microbiome in VTE is a critical step for informing clinical management strategies.

Mendelian randomization (MR) mitigates observational bias by using genetic variation, typically single-nucleotide polymorphisms (SNPs), as instrumental variables (IVs) to establish causal associations between exposures and disease outcomes. The primary advantage of MR approach lies in the random inheritance of genetic variants, which follow Mendel’s laws of segregation and independent assortment, making them less susceptible to environmental confounding.^[11]^ This approach has successfully identified modest risk factors for various diseases, including cancers and cardiovascular diseases.^[12]^ The immune system, with its various cell types and signaling pathways, plays a crucial role in the development of VTE. By analyzing studies that cover 731 immune cell signatures,^[13]^ we can identify specific immune cells that act as mediators linking GMs to VTE risk.

In this study, we performed the MR analysis using publicly available large-scale genome-wide association study (GWAS) data on GMs, immune cells, and VTE. Our results identified specific taxa that either increase or decrease VTE risk via regulating specific immune cells, many of which have not been previously implicated in VTE research. Thus, our findings not only expand the current understanding of GM’s role in VTE but also elucidate the role of immune cells in the progression of VTE, providing a novel approach for the clinical management of the condition.

## 2. Materials and methods

### 2.1. Study design

**Figure 1.**
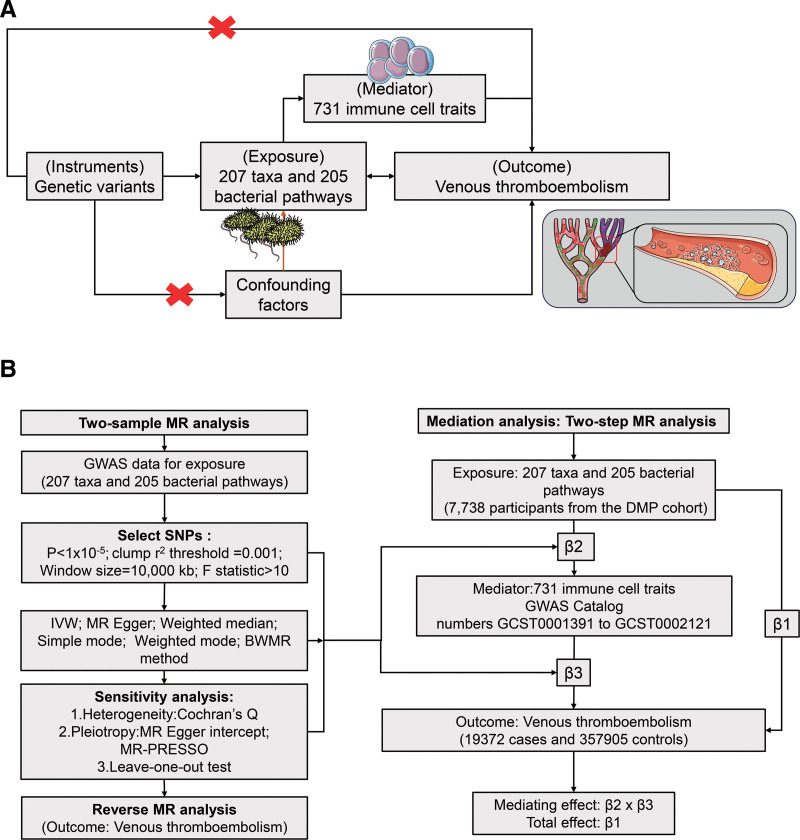
Schematic depiction of the connections between gut microbiota (GM) and venous thromboembolism (VTE) with immune cells as mediators in the bidirectional and mediation Mendelian randomization (MR) analyses. (A) This study utilized a two-sample bidirectional MR approach to examine the causal associations between gut microbiota (exposure) and VTE (outcome). Subsequently, 731 immune cell traits were identified as potential mediators for further mediation analyses. Finally, a two-step MR analysis was conducted to investigate the potential mediating role of immune cells, with step 1 focusing on the impact of gut microbiota on immune cells and step 2 examining the influence of immune cells on VTE. The application of MR in this context relies on 3 fundamental assumptions: assumption 1, the genetic variants must exhibit associations with the exposures; assumption 2, the genetic variants must not demonstrate associations with confounding factors; and assumption 3, the genetic variants must solely influence outcomes through the exposures, excluding alternative pathways. (B) MR analysis flowchart. BWMR = Bayesian-weighted MR, GWAS = genome-wide association study, LD = linkage disequilibrium, MR = Mendelian randomization, SNP = single-nucleotide polymorphism, VTE = venous thromboembolism.

The study was conducted in 2 phases (Fig. 1). First, a two-sample MR approach was used to assess associations between 412 GM components (207 taxa and 205 bacterial pathways) and VTE using SNPs as IVs. In the second phase, potential mediators were selected from 731 immune cell traits to explore their roles in the GM and VTE relationship, with mediating effects quantified using mediation MR analysis. To ensure MR analysis validity, 3 assumptions were required: a robust association between the IVs and exposure, no correlation between the IVs and confounding variables, and the IVs affecting the outcome exclusively through the exposure.^[14]^

### 2.2. Data resources

The data utilized in this study, related to the human gut microbiome, were sourced from publicly available GWAS datasets. All original studies included in these data have obtained ethical approval and informed consent. The gut microbiome GWAS data were obtained from the website (https://www.ebi.ac.uk/gwas/; access codes: GCST90027446 – GCST90027857). Specific access codes for species and pathways are listed in Table S1, Supplemental Digital Content, https://links.lww.com/MD/R388.

We used GWAS data related to immunity to explore the potential mediating role of immune cells. The immune cell data were derived from GWAS summary statistics, covering 731 immune-related whole-genome features (accession numbers: GCST0001391 – GCST0002121).^[13]^ Additionally, the summary data for VTE were obtained from the GWAS summary data repository (https://storage.googleapis.com/finngen-public-data-r9/summary_stats/Finngen_R9_I9_VTE.gz), which includes 19,372 cases of VTE and 357,905 controls. Further details regarding the data used in this MR study are provided in Table 1.

**Table 1:** Details of the genome-wide association studies (GWASs) used in the Mendelian randomization.

Phenotype	Cases/controls or sample sizes	Ancestry	Data source
Exposure			
Gut microbiota	7738	European	http://ftp.ebi.ac.uk/pub/databases/gwas/summary_statistics/ GCST90000001-GCST90001000/ (GCST90027446-GCST90027857)
Outcomes	Cases/controls		
VTE	19,372/357,905	European	https://storage.googleapis.com/finngen-public-data-r9/summary_stats/finngen_R9_I9_VTE.gz
Mediator			
Immune cells	3757	European	http://ftp.ebi.ac.uk/pub/databases/gwas/summary_statistics/GCST90000001-GCST90001000/ (GCST0001391 to GCST0002121)

VTE = venous thromboembolism.

### 2.3. Selection of IVs

We selected independent IVs at a genome-wide significance level of <1.0 × 10^−5^, applying criteria of linkage disequilibrium *r*^2^ < 0.001 and a clumping distance of 10,000 kb. We then calculated the proportion of variation explained (*R*^2^) and used *F*-statistics to assess the instrument strength. The *R*^2^ value represents the proportion of variance in the exposure explained by the IVs, while the *F*-statistic, which quantifies the strength of the IVs-exposure association, is considered robust when exceeding 10.^[15]^ These metrics were computed using the following formulae:


$$\begin{array}{l} {\text{R}}^{\text{2}}=2\times (1-{\text{M}AF)\times MAF\times \beta }^{\text{2}} \end{array}$$

$$\begin{array}{l} F=(R^{2}/(1-R^{2}))\times ((N-k-1)/k), \end{array}$$

where N is the sample size; β is the genetic estimation of each SNP on the exposure; *k* represents the number of SNPs. Minor allele frequency of SNPs is used as IVs.

### 2.4. Two-sample MR and reverse MR analysis

To elucidate the causal relationship between the GMs and VTE, a two-sample MR approach was utilized. The analysis employed the inverse variance-weighted (IVW) and Bayesian-weighted MR (BWMR) methods ^[16]^ to calculate the effects. Continuous results were presented as odds ratios (ORs) with 95% confidence intervals (CIs), while binary outcomes were reported with beta (β) values, standard errors, and corresponding 95% CIs. A threshold of *P* < .05 was applied to determine statistical significance.

To investigate whether VTE exerts a causal influence on the identified GM components (*P*__IVW_ < .05), a reverse MR analysis was conducted. In this analysis, VTE-associated SNPs were utilized as IVs, with VTE serving as the exposure and GM as the outcome. The reverse MR analysis followed a similar procedure to that employed in the standard MR analysis.

### 2.5. Mediation MR analysis

Mediation MR analysis was conducted to explore the exposure to outcome via mediations, thereby elucidating the underlying mechanisms. In this study, the mediation MR analysis was employed to investigate 731 immune cells as potential mediators. First, the causal relationship between GM and immune cells was assessed using MR. Then, the causal relationship between immune cells and VTE was examined. Here, the total effect of GM on VTE is represented as β1, the direct effect of GM on immune cells as β2, and the direct effect of immune cells on VTE as β3. The mediating effect was quantified as the product of β2 and β3.^[17]^

### 2.6. Statistical analysis

To estimate the causal effects between GM exposures and VTE outcome, we employed several high-efficiency methodologies, including the IVW method, weighted median method, weighted mode method, simple mode method, MR-Egger regression, MR-Egger intercept test, Cochrane *Q* test, MR-PRESSO test, and leave-one-out analysis. The IVW method was the primary approach for assessing causal relationships.^[18]^ Heterogeneity among SNPs was evaluated using the Cochran *Q* test.^[19]^ Leave-one-out analysis was conducted to identify heterogeneous SNPs by systematically excluding each instrumental SNP. The MR-Egger regression was used to address potential directional pleiotropy and invalid instruments. When over 50% of the weight was derived from valid instruments, the weighted median estimator was utilized to obtain a reliable estimate.^[20]^ If the hypothesis of instrument strength independent of direct effect is violated, it has been observed that the weighted model estimate exhibits enhanced statistical power in detecting a causal effect, reduced bias, and decreased rates of type I error compared with MR-Egger regression.^[21]^ The MR-Egger intercept *P*-value was used to detect horizontal pleiotropy (*P* < .05 indicating its presence). The MR-PRESSO test identified and excluded outlier SNPs to adjust association results. Associations between the human GM and VTE risk were reported as ORs with 95% CIs. All MR analyses were performed using the “TwoSampleMR” package in R version 4.2.1.

## 3. Results

### 3.1. Main results of the 412 bacterial traits with the risk of VTE

**Figure 2.**
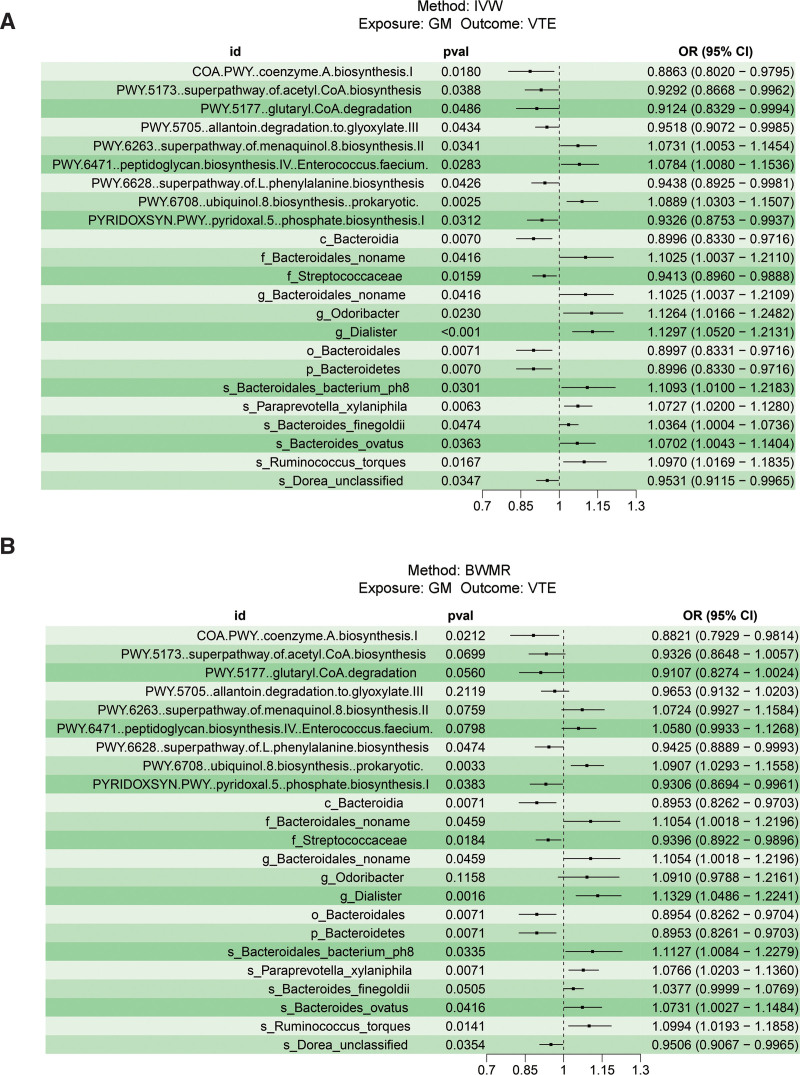
Forest plot of the causal effect of genetically determined bacterial traits on VTE using the IVW and BWMR methods. (A) This figure shows the IVW estimates of significantly VTE-associated gut microbiota taxa. The black bars represent the IVW estimates. (B) This figure shows the BWMR estimates of significantly VTE-associated GMs. The black bars represent the BWMR estimates. The OR > 1 indicates increased risk while <1 indicates decreased risk. BWMR = Bayesian-weighted MR, CI = confidence interval, DVT = deep vein thrombosis, IVW = inverse-variance weighted, OR = odds ratio, PE = pulmonary embolism, SNP = single-nucleotide polymorphism, VTE = venous thromboembolism.

**Figure 3.**
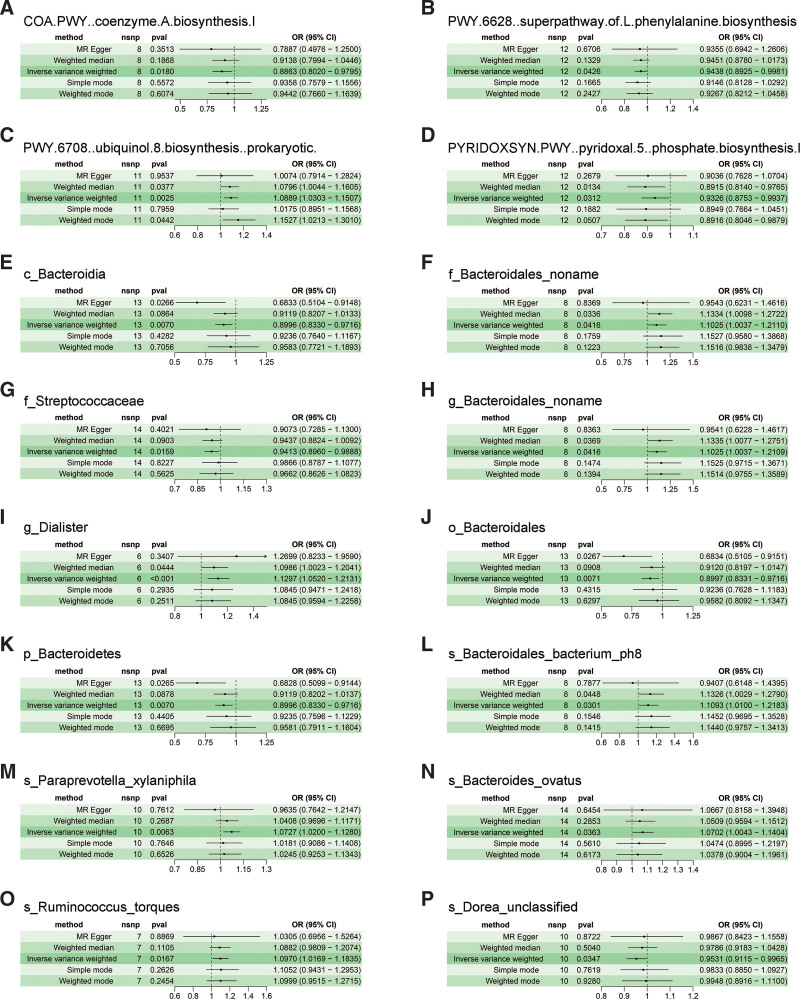
Forest plots showing the causal associations between 11 bacterial traits and VTE by using different methods. (A–P) Forest plot showing the causal effect of taxa and bacterial pathways (exposure) on VTE (outcomes). The OR > 1 indicates increased risk while <1 indicates decreased risk. CI = confidence interval, OR = odds ratio, SNP = single-nucleotide polymorphism, VTE = venous thromboembolism.

A two-sample MR analysis was performed to examine the causal relationship between GM and VTE using the IVW and BWMR methods. The specific IVs utilized for these 412 bacterial traits are listed in Table S2, Supplemental Digital Content, https://links.lww.com/MD/R388. Figure 2A illustrates that the Supplemental Digital Content, https://links.lww.com/MD/R388, IVW model identified 23 VTE-associated GMs. Subsequently, the IVW results were validated using the BWMR method, which identified 16 VTE-related GMs (Fig. 2B). The intersection of IVW and BWMR results was then analyzed using two-sample MR to further investigate 16 GMs and VTEs. According to the results, the Cochran *Q* test indicates no significant heterogeneity (*P* > .05, Table S3, Supplemental Digital Content, https://links.lww.com/MD/R388). The MR-Egger regression intercept method showed no bias due to genetic pleiotropy in the results (Table S4, Supplemental Digital Content, https://links.lww.com/MD/R388). In addition, we found that there was no inverse causal relationship between VTE and 16 GMs (Table S5, Supplemental Digital Content, https://links.lww.com/MD/R388). Table S6, Supplemental Digital Content, https://links.lww.com/MD/R388, contains specific 204 SNPs associated with VTE. Based on the above screening criteria, we ultimately screened a total of 16 GMs for MR analysis. Figure 3 outlined an additional investigation into the causal relationship between 16 GMs and VTE. In the MR-PRESSO analysis, the effect estimates of associations between 16 bacterial traits and VTE were obtained, with all traits demonstrating a lack of horizontal pleiotropy (Table S7, Supplemental Digital Content, https://links.lww.com/MD/R388).

Based on the findings presented in Figure 3, to further ensure the robustness and consistency of causal effects, we selected microbiota for which the directions of the MR-Egger and IVW methods were aligned for subsequent analysis (excluding 4 specific bacterial traits, namely “f_Bacteroidales_noname,” “g_Bacteroidales_noname,” “s_Bacteroidales_bacterium_ph8,” and “s_Paraprevotella_xylaniphila”). We integrated the following sensitivity analysis approaches to comprehensively assess the reliability of the causal effects between the remaining 12 bacterial traits and VTE. Additionally, the stability of the results was further corroborated by scatter plots (Fig. S1, Supplemental Digital Content, https://links.lww.com/MD/R387), forest plots (Fig. S2, Supplemental Digital Content, https://links.lww.com/MD/R387), and funnel plots (Fig. S3, Supplemental Digital Content, https://links.lww.com/MD/R387). Moreover, analyses employing the “leave-one-out” method revealed that no individual variable exerted a significant influence on the causal inferences (Fig. S4, Supplemental Digital Content, https://links.lww.com/MD/R387).

### 3.2. Causal effects between GMs and 731 immune cell traits

**Figure 4.**
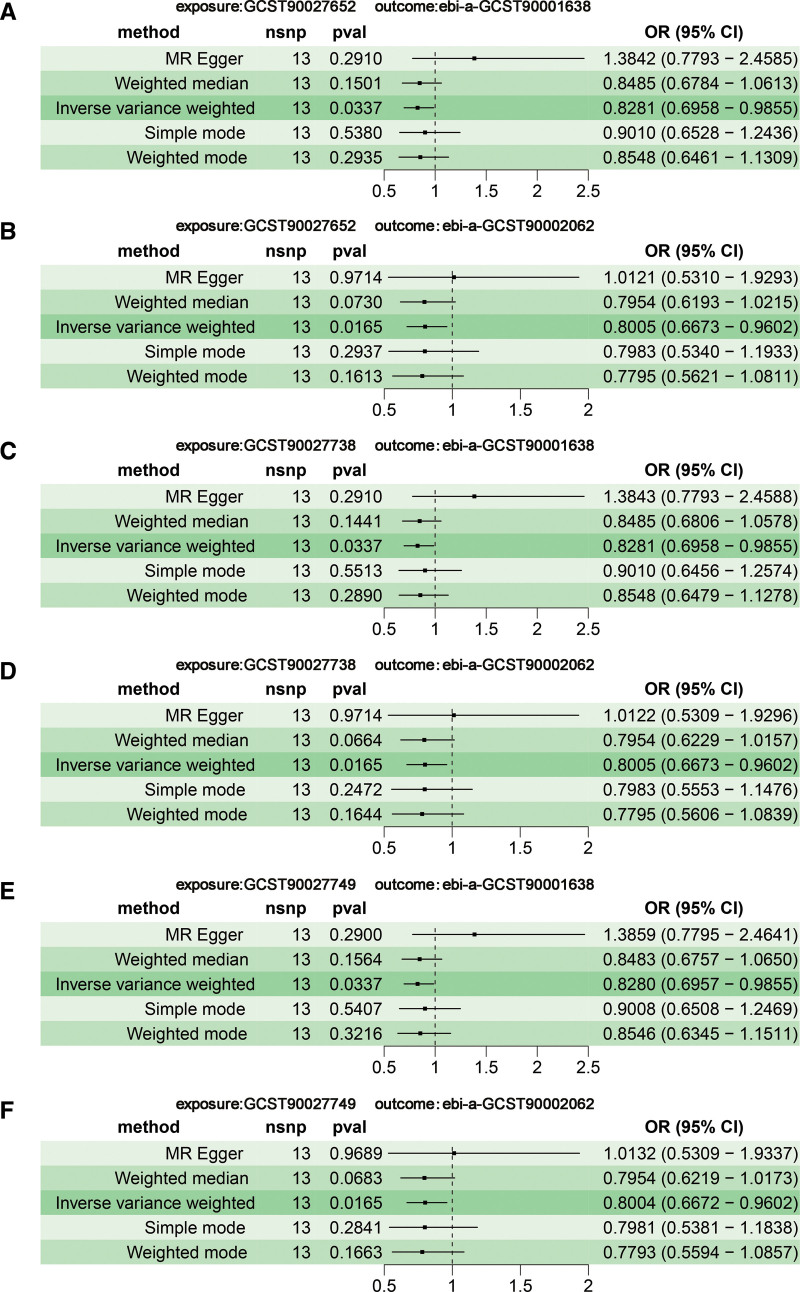
Forest plots showing the causal associations between 3 bacterial traits and 6 immune cell traits by using different methods. (A–F) The causal connections between 3 taxa and immune cell traits in MR analyses. The OR > 1 indicates increased risk while <1 indicates decreased risk. CI = confidence interval, OR = odds ratio, SNP = single-nucleotide polymorphism.

The intricate relationship between the GM within the human body and immune cells is inextricable. Following this, we investigated how GM influences the characteristics of immune cells and its potential impact on VTE risk. In this study, a mediated MR analysis, involving 731 immune cell traits (Table S8, Supplemental Digital Content https://links.lww.com/MD/R388), was employed to explore the connection between GMs and VTE. To ensure a robust mediating role of immune cells in bacterial traits, we conducted a screening process for the bacterial traits included in our study. By integrating MR-Egger, IVW, and BWMR methods (*P* < .05), we identified 3 bacterial traits (namely c_Bacteroidia, o_Bacteroidales, and p_Bacteroidetes). Then, the significant associations were identified between 3 bacterial traits and various immune cell traits. Of 731 potential mediators, 66 immune cell characteristics met the screening criteria (*P*__IVW_ < .05) and were subsequently included in the mediation MR analysis (Table S9, Supplemental Digital Content, https://links.lww.com/MD/R388). Subsequent investigations explored the potential mediating effects of these significant mediators (66 immune cell traits derived from 3 bacterial traits) exposures on VTE. We first performed a two-sample MR analysis using the IVW (*P* < .05) method to examine the causal relationship between 66 immune cells and VTE, in which there were 2 types of immune cells related to the regulation of bacterial traits, both “DN (CD4^−^ CD8^−^) NKT % lymphocyte” (ebi-a-GCST90001638) and “CD4 on CD28^+^ CD4^+^” (ebi-a-GCST90002062). IVW (Fig. 4) analysis indicated that 3 GMs were found to impact VTE through 6 immune cell traits.

After examining the impact of immune cell characteristics on VTE, we subsequently investigated the direct effects of 3 GMs on these critical mediators. The causal relationship between the 3 GMs and the 6 immune cell traits was assessed using a two-sample MR analysis. The MR-Egger intercept method results showed that genetic pleiotropy did not affect the findings (Table S10, Supplemental Digital Content, https://links.lww.com/MD/R388). Cochran *Q* tests indicated the absence of significant heterogeneity (*P* > .05; Table S11, https://links.lww.com/MD/R388), and MR-PRESSO analysis confirmed no evidence of horizontal pleiotropy (*P* > .05; Table S12, Supplemental Digital Content, https://links.lww.com/MD/R388). Furthermore, the consistency of the results was substantiated by scatter plots (Fig. S5, Supplemental Digital Content, https://links.lww.com/MD/R387), forest plots (Fig. S6, Supplemental Digital Content, https://links.lww.com/MD/R387), funnel plots (Fig. S7, Supplemental Digital Content, https://links.lww.com/MD/R387), and “leave-one-out” analyses (Fig. S8, Supplemental Digital Content, https://links.lww.com/MD/R387).

The IVW approach was employed to examine the impact of 6 different immunophenotypes on VTE risk, which include “(from c_Bacteroidia) DN (CD4^−^ CD8^−^) NKT %lymphocyte,” “(from c_Bacteroidia) CD4 on CD28^+^ CD4^+^” cells, “(from o_Bacteroidales) DN (CD4^−^ CD8^−^) NKT %lymphocyte,” “(from o_Bacteroidales) CD4 on CD28^+^ CD4^+^” cells, “(from p_Bacteroidetes) DN (CD4^−^ CD8^−^) NKT %lymphocyte,” and “(from p_Bacteroidetes) CD4 on CD28^+^ CD4^+^” cells. Four more techniques, MR eager, weighted median, simple mode, and weighted mode, produced similar outcomes (Fig. S9, Supplemental Digital Content, https://links.lww.com/MD/R387).

Additionally, both the MR-Egger intercept analysis (Table S13, Supplemental Digital Content, https://links.lww.com/MD/R388) and the MR-PRESSO analysis (Table S14, Supplemental Digital Content, https://links.lww.com/MD/R388) provided evidence against horizontal pleiotropy in the studied associations. Cochran *Q* tests revealed no significant heterogeneity among the results (*P* > .05; Table S15, https://links.lww.com/MD/R388). The robustness of the findings was further corroborated by scatter diagrams (Fig. S10, Supplemental Digital Content, https://links.lww.com/MD/R387), forest plots (Fig. S11, Supplemental Digital Content, https://links.lww.com/MD/R387), funnel plots (Fig. S12, Supplemental Digital Content, https://links.lww.com/MD/R387), and “leave-one-out” analyses (Fig. S13, https://links.lww.com/MD/R387), collectively reinforcing the reliability of the study outcomes.

### 3.3. Mediation MR analysis

A two-step MR study elucidated a causal pathway from GM to VTE, mediated by immune cell traits. The impact of key GM exposures on VTE was quantified, with mediation effects detailed in Table 2. For instance, the bacterial class “c_Bacteroidia” was found to inhibit VTE progression through interactions with specific immune cell traits. Specifically, “c_Bacteroidia” interacted with “DN (CD4^−^ CD8^−^) NKT %lymphocyte,” yielding a total effect size of −0.1057551, with a mediated effect size of 0.006648427 attributed to this immune cell trait. Additionally, “c_Bacteroidia” hindered VTE via mediation by “CD4 on CD28^+^ CD4^+^” cells, with a total effect size of -0.1057551 and a mediated effect size of −0.006887326 for these cells. These results underscore the complex interplay between distinct GM exposures, their immune-mediated pathways, and their cumulative impact on VTE.

**Table 2:** Mediation Mendelian randomization analyses of the causal effects between gut microbiota, immune cells, and VTE.

Exposure	Exposure (ID)	Mediator	Mediator (ID)	Total effect	β1	β2	Indirect effect (β1*β2)
c_Bacteroidia	GCST90027652	CD4^−^ CD8- NKT	ebi-a-GCST90001638	−0.1,057,551	−0.18,861	−0.03525	0.006648427
c_Bacteroidia	GCST90027652	CD4 on CD28^+^ CD4^+^ Treg	ebi-a-GCST90002062	−0.1,057,551	−0.22,258	0.03094	−0.006887326
o_Bacteroidales	GCST90027738	CD4^−^ CD8-NKT	ebi-a-GCST90001638	−0.1,057,279	−0.18,861	−0.03525	0.006648554
o_Bacteroidales	GCST90027738	CD4 on CD28^+^ CD4^+^ Treg	ebi-a-GCST90002062	−0.1,057,279	−0.22,258	0.03094	−0.006887242
p_Bacteroidetes	GCST90027749	CD4^−^ CD8-NKT	ebi-a-GCST90001638	−0.1,057,868	−0.1887	−0.03525	0.006651576
p_Bacteroidetes	GCST90027749	CD4 on CD28^+^ CD4^+^ Treg	ebi-a-GCST90002062	−0.1,057,868	−0.22,262	0.03094	−0.006888577

VTE = venous thromboembolism.

## 4. Discussion

This study is one of the first to comprehensively explore the causal links between GM, immune cells, and VTE via MR analysis. Observational studies show a correlation between GM and VTE. Gut microbiome disruptions can activate inflammatory pathways in vascular cells, platelets, and immune cells, leading to a prothrombotic state.^[22]^ Thrombosis development involves complex interactions among coagulation, immunity, and inflammation, with dysbiosis, increased intestinal permeability, and metabolite production playing key roles. Our study identified 12 taxa and 4 bacterial metabolic pathways associated with VTE risk, with 3 taxa influencing VTE risk via immune cell effects.

Statins, such as atorvastatin, can reduce serum cholesterol levels and potentially lower coronary heart disease risk by decreasing serum ubiquinol and cholesterol.^[23]^ However, we found that the “PWY.6708-ubiquinol 8 biosynthesis prokaryotic” pathways (*P_*_IVW_: .0025, OR: 1.0889) may increase VTE risk, although this causal relationship needs further experimental validation. The tricarboxylic acid cycle and lipid synthesis both rely on coenzyme A (CoA) as a cofactor.^[24]^ While we understand much about the sources and regulation of CoA’s acyl groups, its broader cellular roles and biosynthesis still need further study. Dysregulation of CoA has also been linked to cardiovascular events. Our studies showed that the “COA. PWY-coenzyme A biosynthesis I” pathway (*P*__IVW_: .0180, OR: 0.8863) may reduce VTE incidence, but the specific mechanisms behind this effect require more investigation. L-phenylalanine, an aromatic amino acid produced by enterobacteria, is linked to cardiovascular risk.^[25]^ It is metabolized by gut microbes into phenylacetic acid, then converted to phenylacetylglutamine, which affects thrombosis.^[26]^ We found that although the “PWY.6628-superpathway of L phenylalanine biosynthesis” pathway (*P_*_IVW_: .0426, OR: 0.9438) is negatively correlated with VTE risk, the underlying mechanisms, especially the roles of related metabolites, require further investigation. Vitamin B6 (pyridoxal 5’-phosphate) is a key cofactor in biochemical reactions and catabolic pathways.^[27]^ Hyperhomocysteinemia (HHcy) is a cardiovascular risk factor,^[28]^ and vitamin B6 deficiency can induce HHcy. Studies show HHcy causes endothelial dysfunction and atherosclerosis. Our study showed that the “PYRIDOXSYN.PWY-pyridoxal 5 phosphate biosynthesis I” pathway (*P_*_IVW_: .0312, OR: 0.9326) can reduce VT risk.

Furthermore, through the IVW and BWMR method (both *P_*_IVW_ and *P_*_BWMR_ < .05), we have identified 7 taxa (f_Bacteroidales_noname, g_Bacteroidales_noname, g_Dialister, s_Bacteroidales_bacterium_ph8, s_Paraprevotella_xylaniphila, s_Bacteroides_ovatus, s_Ruminococcus_torques) positively correlated with a causal relationship to VTE, and 5 bacterial species (c_Bacteroidia, f_Streptococcaceae, o_Bacteroidales, p_Bacteroidetes, s_Dorea_unclassified) negatively correlated with a causal relationship. These findings suggest that identifying specific bacterial strains in fecal samples could be a prognostic biomarker and a potential target for effective VTE interventions. The GM regulates immunity, and its imbalance can disrupt immune cell function and increase disease risk, including VTE. We chose 3 bacteria (c_Bacteroidia, o_Bacteroidales, p_Bacteroidetes) as exposure factors because their *P*-values were <.05 in both IVW and MR-Egger methods. This dual criterion ensures the robustness of the causal relationship and the stability of immune cells as mediators. The Bacteroidetes phylum is a dominant gut bacterial group in healthy individuals.^[29]^ They perform key metabolic functions in the colon.^[30]^ As obligate anaerobes, Bacteroidetes have a complex cell wall structure and generate energy through glycolysis. They excel at breaking down dietary fiber and, in its absence, can metabolize host-produced mucins.^[31]^ Additionally, they degrade dietary or host-derived proteins, especially in low-carbohydrate diets, producing nitrogen-containing metabolites.^[32]^ Interestingly, we found that “c_Bacteroidia,” “o_Bacteroidales,” and “p_Bacteroidetes” were discovered to reduce the likelihood of VTE by affecting “DN (CD4^−^ CD8^−^) NKT %lymphocyte” and “CD4 on CD28^+^ CD4^+^” cells (Table 2). However, further exploration is needed on the regulation of immune cells by this metabolic pathway. Natural killer T cells (NKT cells) are a unique T cell subset with both T cell receptors and NK cell receptors on their surface.^[33]^ They produce large amounts of cytokines and have cytotoxic functions similar to NK cells. Human NKT cells can be CD4^+^ CD8^−^, CD4^−^ CD8^+^, or double-negative CD4^−^ CD8^−^ cells, but the origin of CD4^−^ CD8^−^(DN) NKT cells is still unclear.^[34]^ CD28 is a key member of the costimulatory molecule subfamily. It provides a “second signal” that lowers the threshold for T cell activation and enhances the initial activation signal.^[35]^ CD28 engagement induces epigenetic, transcriptional, and posttranslational changes in T cells, promoting their development and proliferation. However, many aspects of CD28 biology remain unclear. Regulatory T cells are crucial for immune tolerance and homeostasis.^[36]^ They self-regulate via CTLA-4 and CD28-dependent feedback loops.^[37]^ Recent findings challenge the notion that regulatory cells are always pathogenic, supporting the idea of lineage plasticity. Regulatory cell stability is a major concern in therapeutic applications. We showed that “CD4 on CD28^+^ CD4^+^” regulatory cells help inhibit VTE risk under the influence of specific GM.

Understanding the role of specific gut microbes in VTE could enhance preventive and management strategies. However, no MR studies have explored the mediating effect of immune cells in the GM on VTE risk. Such research is crucial for developing effective public health policies and clinical interventions to reduce VTE incidence and societal impact. Our analysis offers several advantages: it robustly addresses causality and confounding variables, covers a broad population sample at low cost, and provides the first genetic evidence linking GM, immune cells, and VTE. The *F*-statistics of our IVs exceeded 10, minimizing weak instrument bias. Future research will focus on further investigating these causal associations. Our findings can guide the selection of specific gut bacteria for deeper investigation into VTE pathogenesis.

However, this study has several limitations. First, bacterial taxa analysis was limited to the genus level, without exploring species or strains. Second, most participants were of European descent, which may limit the generalizability of the findings to other ethnic groups. Third, we used a significance threshold of *P* < 1.0 × 10^−5^ for selecting GM IVs, higher than the conventional genome-wide significance level of *P* < 5 × 10^−8^. We used MR analysis to analyze immune cells as mediators and concluded a causal relationship between GMs and VTE, but we should pay attention to the limitations of these analysis methods, genetic variation is generally less influential on most risk factors, and the variability explained is limited, which may lead to the risk of low statistical power and false negative results. Fourth, information on VTE subtypes was unavailable, necessitating further investigations when such data become available. Finally, although we incorporated immune cells as mediators in the causal relationship between GM and VTE for the first time, our analysis revealed that their mediating effect was not substantial. This suggests that other mechanisms may be at play and further longitudinal studies and mechanistic experiments are needed to verify the specific role of immune cell involvement.

## 5. Conclusion

In summary, this study leveraged publicly available genetic databases to establish causal links between specific gut microbes and VTE. It also highlights the role of the GM in immune responses and its potential impact on VTE. These findings could advance our understanding of VTE pathogenesis and inform novel treatment strategies.

## Acknowledgments

We would like to thank all the participants and researchers who contributed to the GWAS summary-level information public. An earlier version of this paper has been presented as a preprint according to the following link https://www.researchsquare.com/article/rs-3284945/v1.

## Author contributions

**Conceptualization:** Chao Wang, Xiaofa Wu, Jia Wang.

**Data curation:** Chao Wang, Xiaofa Wu, Jia Wang.

**Formal analysis:** Chao Wang, Xiaofa Wu, Jia Wang.

**Funding acquisition:** Chao Wang.

**Investigation:** Chao Wang.

**Resources:** Chao Wang.

**Software:** Chao Wang, Xiaofa Wu.

**Visualization:** Chao Wang.

**Project administration:** Bojian Fei, Hui Jiang.

**Validation:** Bojian Fei, Hui Jiang.

**Supervision:** Hui Jiang.

**Writing – original draft:** Chao Wang, Xiaofa Wu, Jia Wang.

**Writing – review & editing:** Bojian Fei, Hui Jiang.

## Supplementary Material

## References

[1] Khan F Tritschler T Kahn SR Rodger MA. Venous thromboembolism. Lancet. 2021;398:64–77. 33984268 10.1016/S0140-6736(20)32658-1

[2] Duffett L. Deep venous thrombosis. Ann Intern Med. 2022;175:ITC129–44. 36095313 10.7326/AITC202209200

[3] Watson T Shantsila E Lip GYH. Mechanisms of thrombogenesis in atrial fibrillation: Virchow’s triad revisited. Lancet. 2009;373:155–66. 19135613 10.1016/S0140-6736(09)60040-4

[4] Feng Q Chen WD Wang YD. Gut microbiota: an integral moderator in health and disease. Front Microbiol. 2018;9:151. 29515527 10.3389/fmicb.2018.00151 PMC5826318

[5] Furusawa Y Obata Y Hase K. Commensal microbiota regulates T cell fate decision in the gut. Semin Immunopathol. 2015;37:17–25. 25315350 10.1007/s00281-014-0455-3

[6] Michaudel C Sokol H. The gut microbiota at the service of immunometabolism. Cell Metab. 2020;32:514–23. 32946809 10.1016/j.cmet.2020.09.004

[7] He M Tan CP Xu YJ Liu Y. Gut microbiota-derived trimethylamine-N-oxide: a bridge between dietary fatty acid and cardiovascular disease? Food Res Int. 2020;138(Pt B):109812. 33288187 10.1016/j.foodres.2020.109812

[8] Zhu W Gregory JC Org E. Gut microbial metabolite TMAO enhances platelet hyperreactivity and thrombosis risk. Cell. 2016;165:111–24. 26972052 10.1016/j.cell.2016.02.011 PMC4862743

[9] Grimnes G Bhoelan S Hindberg K. Impact of a vancomycin-induced shift of the gut microbiome in a gram-negative direction on plasma factor VIII: C levels: results from a randomized controlled trial. Thromb Haemost. 2022;122:540–51. 34428832 10.1055/s-0041-1733906

[10] Perler BK Friedman ES Wu GD. The role of the gut microbiota in the relationship between diet and human health. Annu Rev Physiol. 2023;85:449–68. 36375468 10.1146/annurev-physiol-031522-092054

[11] Sleiman PMA Grant SFA. Mendelian randomization in the era of genomewide association studies. Clin Chem. 2010;56:723–8. 20224045 10.1373/clinchem.2009.141564

[12] Nordestgaard AT. Causal relationship from coffee consumption to diseases and mortality: a review of observational and Mendelian randomization studies including cardiometabolic diseases, cancer, gallstones and other diseases. Eur J Nutr. 2022;61:573–87. 34319429 10.1007/s00394-021-02650-9

[13] Orrù V Steri M Sidore C. Complex genetic signatures in immune cells underlie autoimmunity and inform therapy. Nat Genet. 2020;52:1036–45. 32929287 10.1038/s41588-020-0684-4 PMC8517961

[14] Didelez V Sheehan N. Mendelian randomization as an instrumental variable approach to causal inference. Stat Methods Med Res. 2007;16:309–30. 17715159 10.1177/0962280206077743

[15] Burgess S Thompson SG. Bias in causal estimates from Mendelian randomization studies with weak instruments. Stat Med. 2011;30:1312–23. 21432888 10.1002/sim.4197

[16] Zhao J Ming J Hu X Chen G Liu J Yang C. Bayesian weighted Mendelian randomization for causal inference based on summary statistics. Bioinformatics. 2020;36:1501–8. 31593215 10.1093/bioinformatics/btz749

[17] Wang C Wang J Guan W Fei B. Impact the impact of gut microbiota on gastric cancer via immune cells: a comprehensive Mendelian randomization study and mediation analysis. Discov Oncol. 2024;15:389. 39215888 10.1007/s12672-024-01285-6 PMC11365895

[18] Bowden J Del Greco M F Minelli C Davey Smith G Sheehan N Thompson J. A framework for the investigation of pleiotropy in two-sample summary data Mendelian randomization. Stat Med. 2017;36:1783–802. 28114746 10.1002/sim.7221 PMC5434863

[19] Hoaglin DC. Misunderstandings about *Q* and ‘Cochran *Q* test’ in meta-analysis. Stat Med. 2016;35:485–95. 26303773 10.1002/sim.6632

[20] Bowden J Davey Smith G Haycock PC Burgess S. Consistent estimation in Mendelian randomization with some invalid instruments using a weighted median estimator. Genet Epidemiol. 2016;40:304–14. 27061298 10.1002/gepi.21965 PMC4849733

[21] Hartwig FP Davey Smith G Bowden J. Robust inference in summary data Mendelian randomization via the zero modal pleiotropy assumption. Int J Epidemiol. 2017;46:1985–98. 29040600 10.1093/ije/dyx102 PMC5837715

[22] Hasan RA Koh AY Zia A. The gut microbiome and thromboembolism. Thromb Res. 2020;189:77–87. 32192995 10.1016/j.thromres.2020.03.003 PMC8780211

[23] Mabuchi H Higashikata T Kawashiri M. Reduction of serum ubiquinol-10 and ubiquinone-10 levels by atorvastatin in hypercholesterolemic patients. J Atheroscler Thromb. 2005;12:111–9. 15942122 10.5551/jat.12.111

[24] Barritt SA DuBois-Coyne SE Dibble CC. Coenzyme A biosynthesis: mechanisms of regulation, function and disease. Nat Metab. 2024;6:1008–23. 38871981 10.1038/s42255-024-01059-y

[25] Qiu M Jiang J Jiang W. The biosynthesis of L-phenylalanine-derived compounds by engineered microbes. Biotechnol Adv. 2024;77:108448. 39260779 10.1016/j.biotechadv.2024.108448

[26] Sulaiman D Reddy ST Fogelman AM. Evidence further linking the intestine to cardiovascular disease. Curr Opin Lipidol. 2024;35:223–7. 39073599 10.1097/MOL.0000000000000944 PMC11377042

[27] Ueland PM McCann A Midttun O Ulvik A. Inflammation, vitamin B6 and related pathways. Mol Aspects Med. 2017;53:10–27. 27593095 10.1016/j.mam.2016.08.001

[28] Austin RC Lentz SR Werstuck GH. Role of hyperhomocysteinemia in endothelial dysfunction and atherothrombotic disease. Cell Death Differ. 2004;11(Suppl 1):S56–64. 15243582 10.1038/sj.cdd.4401451

[29] Ley RE Turnbaugh PJ Klein S Gordon JI. Microbial ecology: human gut microbes associated with obesity. Nature. 2006;444:1022–3. 17183309 10.1038/4441022a

[30] David LA Maurice CF Carmody RN. Diet rapidly and reproducibly alters the human gut microbiome. Nature. 2014;505:559–63. 24336217 10.1038/nature12820 PMC3957428

[31] Flint HJ Scott KP Duncan SH Louis P Forano E. Microbial degradation of complex carbohydrates in the gut. Gut Microbes. 2012;3:289–306. 22572875 10.4161/gmic.19897 PMC3463488

[32] Kapoor P Tiwari A Sharma S. Effect of anthocyanins on gut health markers, Firmicutes-Bacteroidetes ratio and short-chain fatty acids: a systematic review via meta-analysis. Sci Rep. 2023;13:1729. 36720989 10.1038/s41598-023-28764-0 PMC9889808

[33] Crosby CM Kronenberg M. Tissue-specific functions of invariant natural killer T cells. Nat Rev Immunol. 2018;18:559–74. 29967365 10.1038/s41577-018-0034-2 PMC6343475

[34] Hu Z Gu W Wei Y Liu G Wu S Liu T. NKT cells in mice originate from cytoplasmic CD3-positive, CD4-CD8- double-negative thymocytes that express CD44 and IL-7Rα. Sci Rep. 2019;9:1874. 30755654 10.1038/s41598-018-37811-0 PMC6372634

[35] Esensten JH Helou YA Chopra G Weiss A Bluestone JA. CD28 costimulation: from mechanism to therapy. Immunity. 2016;44:973–88. 27192564 10.1016/j.immuni.2016.04.020 PMC4932896

[36] Göschl L Scheinecker C Bonelli M. Treg cells in autoimmunity: from identification to Treg-based therapies. Semin Immunopathol. 2019;41:301–14. 30953162 10.1007/s00281-019-00741-8

[37] Marangoni F Zhakyp A Corsini M. Expansion of tumor-associated Treg cells upon disruption of a CTLA-4-dependent feedback loop. Cell. 2021;184:3998–4015.e19. 34157302 10.1016/j.cell.2021.05.027 PMC8664158

