# Supplementary material for: Investigating the association between gut microbiota and venous thromboembolism mediated by immune cells: A mediation Mendelian randomization study
Source: Medicine (Baltimore). 2026 Feb 20;105(8):e47709. doi: 10.1097/MD.0000000000047709 (PMC12928951; doi:10.1097/MD.0000000000047709)
Supplement: Supplementary file 2 [file medi-105-e47709-s002.docx]

**Supplementary figure**

*Figure S1 Scatter plots for the causal association between 12 bacterial traits and VTE*

*
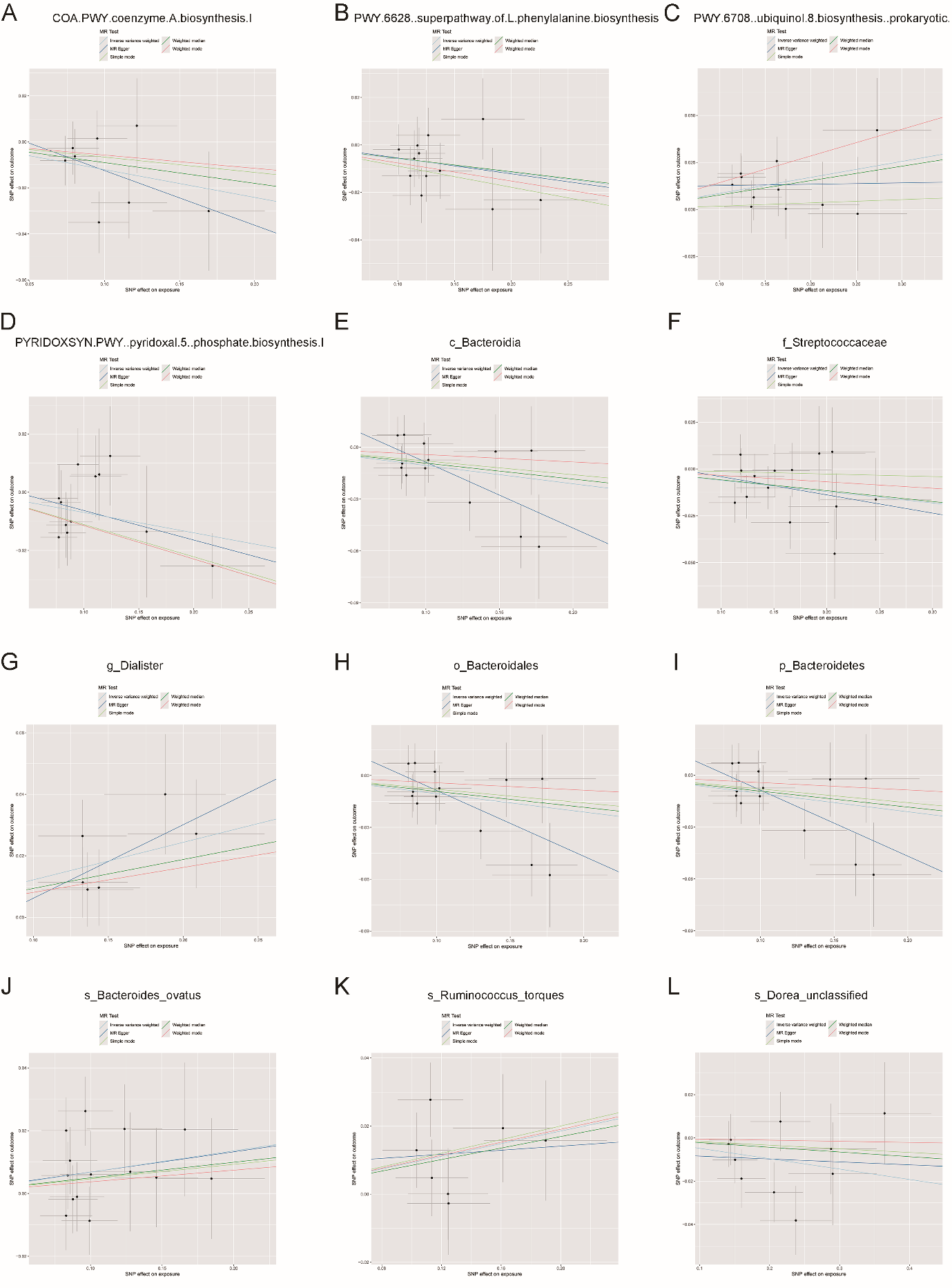
*

*Figure S2 Forest plot of the causal effect of 12 bacterial traits on VTE*

*
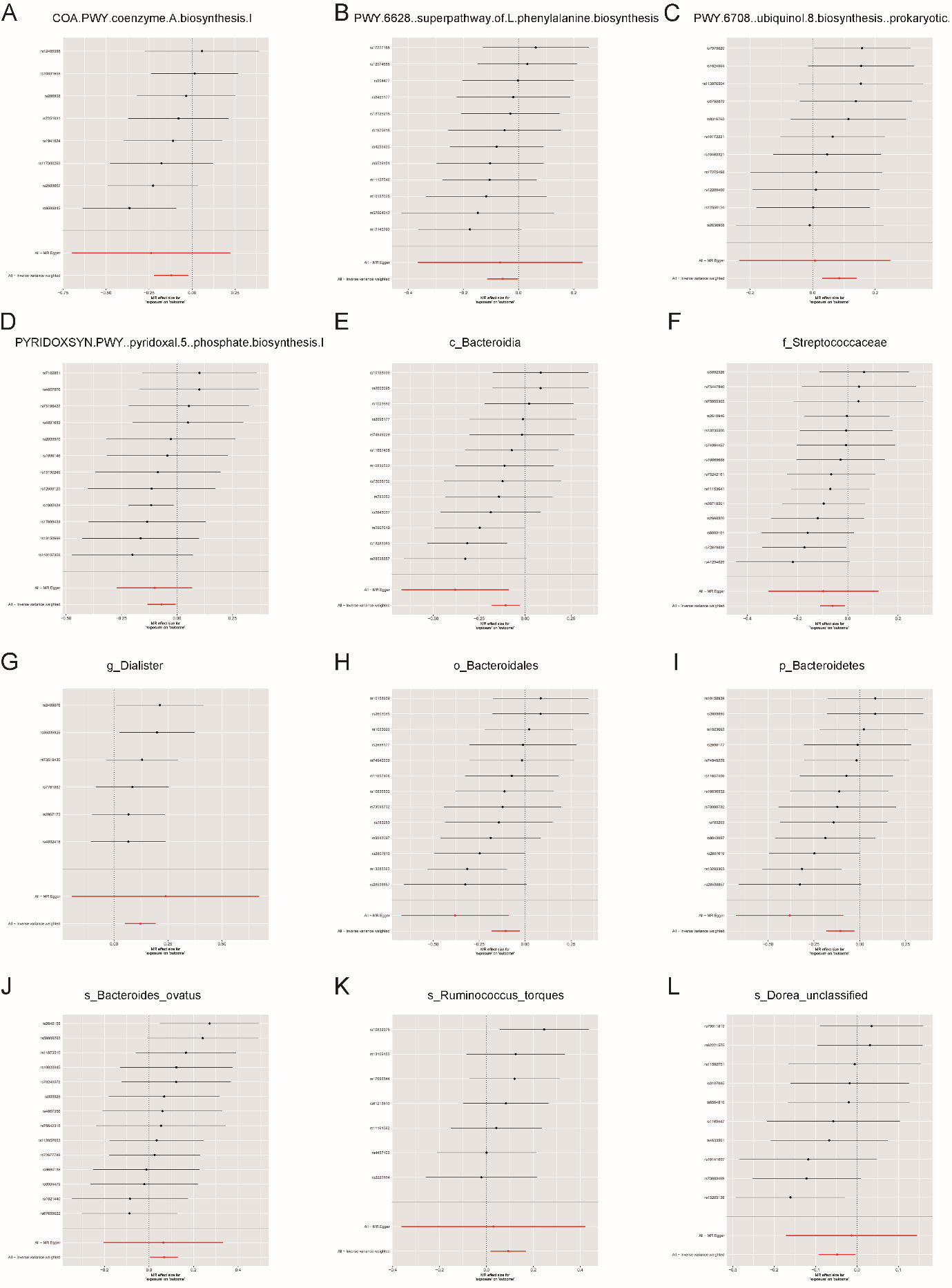
*

*Figure S3 Funnel plot of the causal effect of 12 bacterial traits on VTE*

*
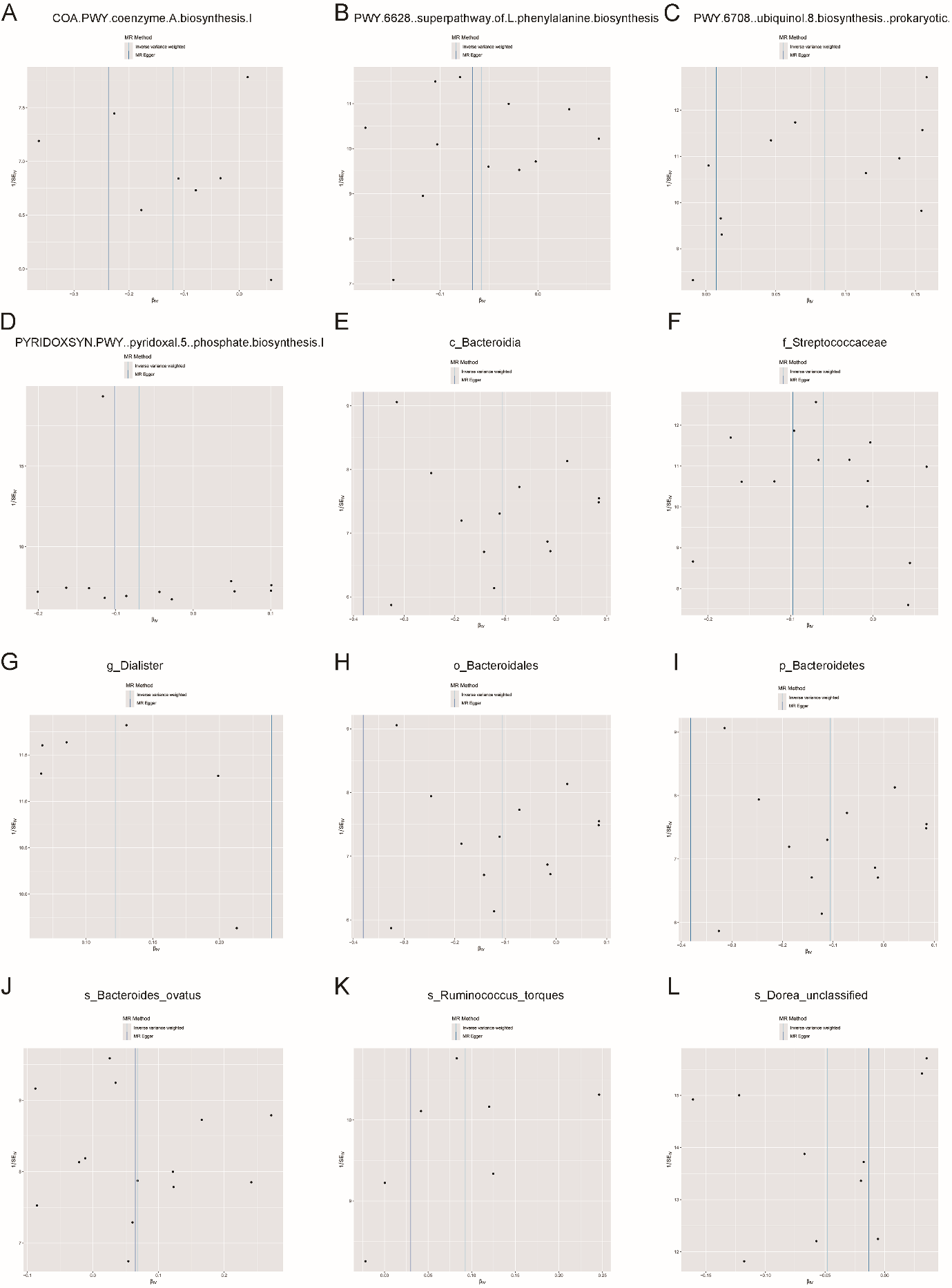
*

*Figure S4 Leave-one-out plots for the causal association between 12 bacterial traits and VTE*

*
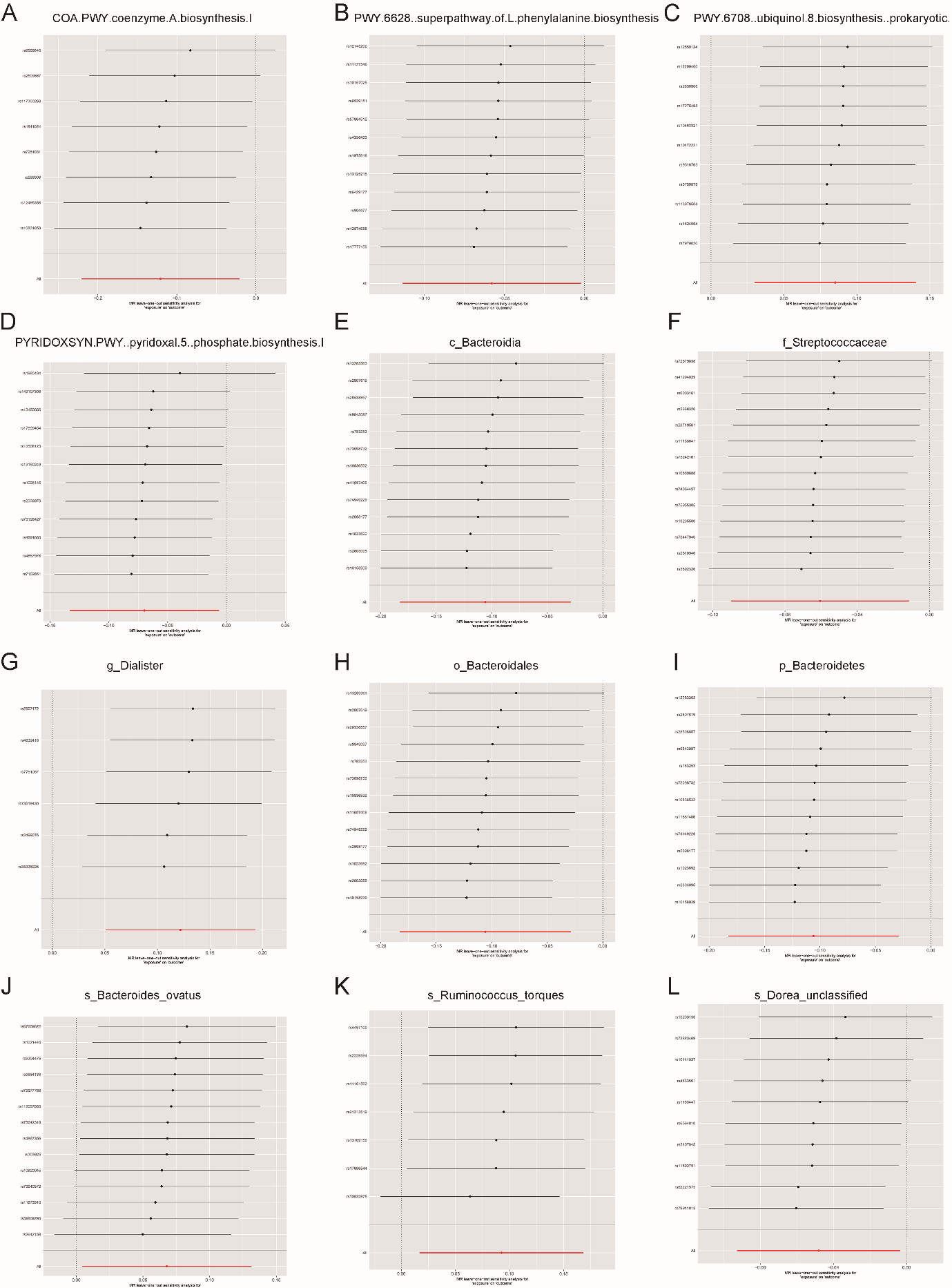
*

*Figure S5 Scatter plots for the causal association between the three GMs and the six immune cell traits*

*
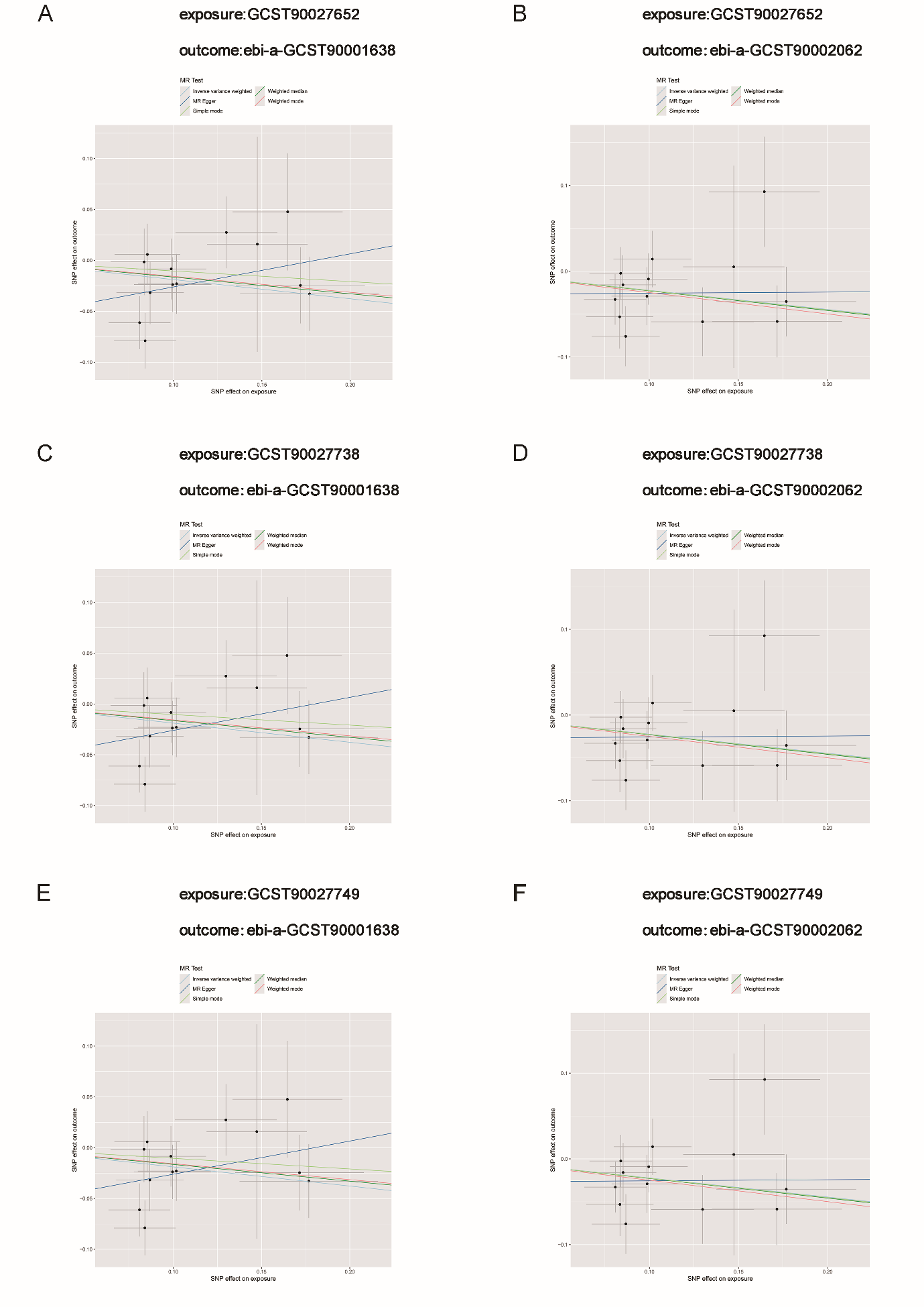
*

*Figure S6 Forest plot of the causal effect of the three GMs on the six immune cell traits*

*
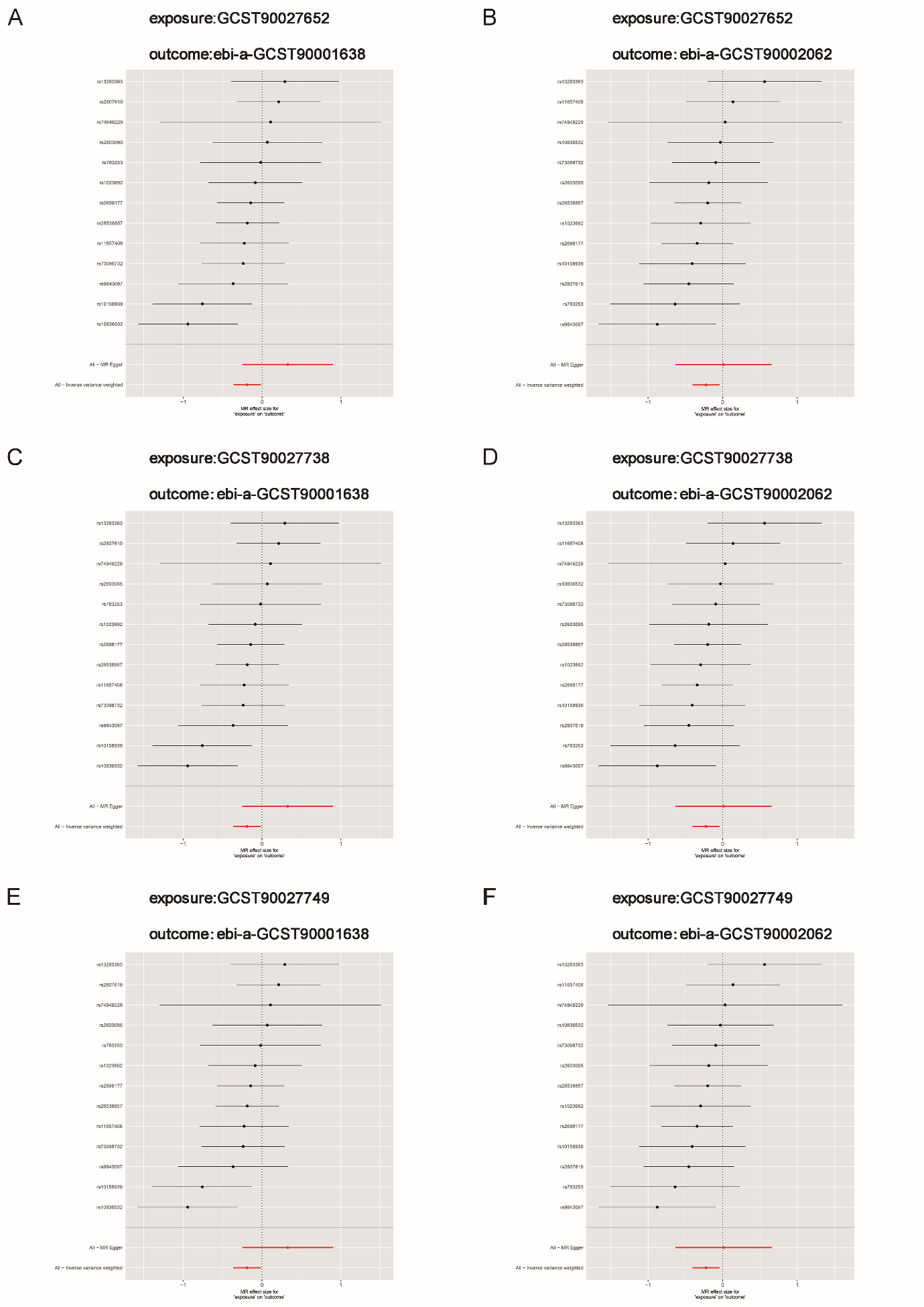
*

*Figure S7 Funnel plot of the causal effect of the three GMs on the six immune cell traits*

*
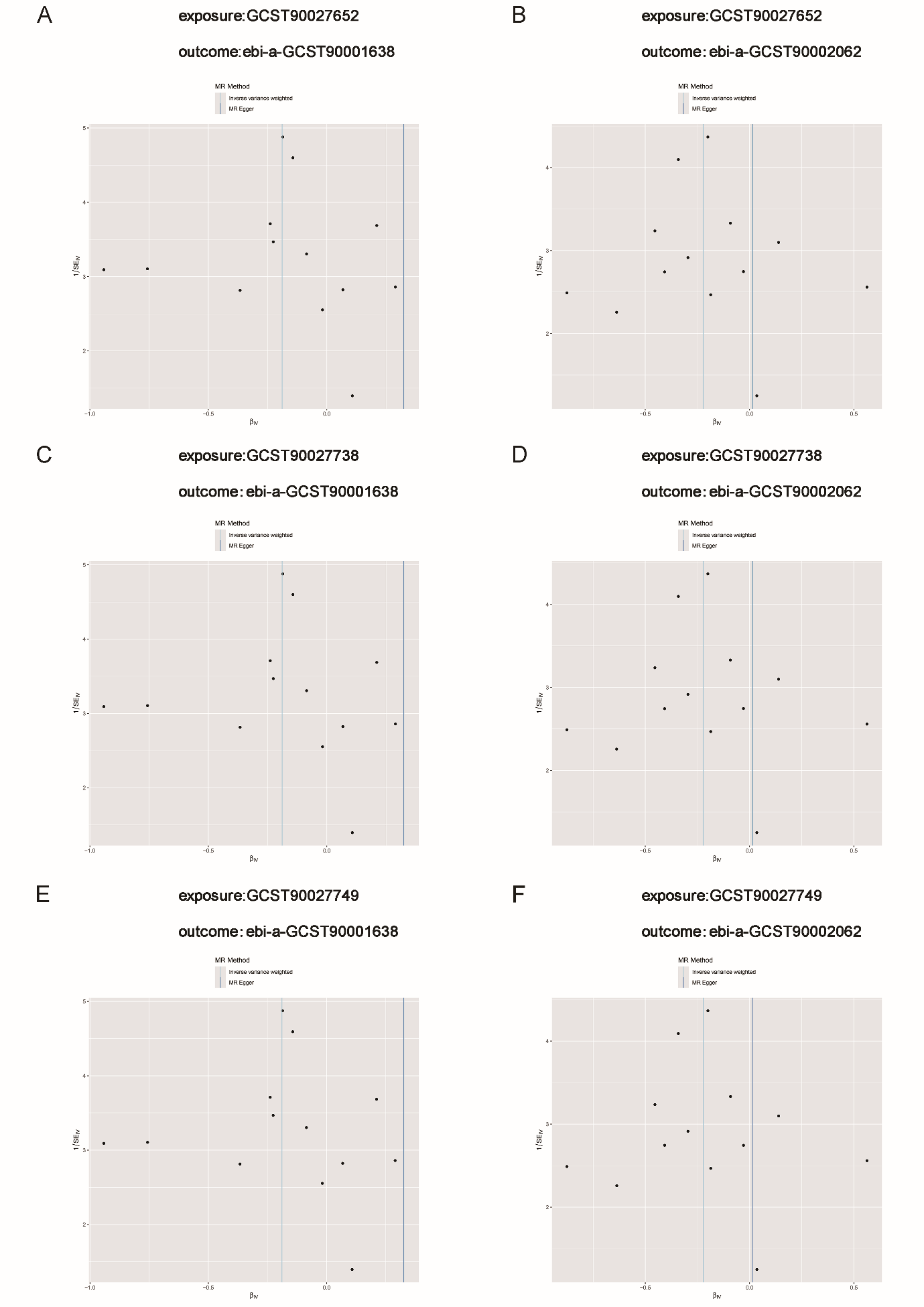
*

*Figure S8 Leave-one-out plots for the causal association between the three GMs and the six immune cell traits*

*
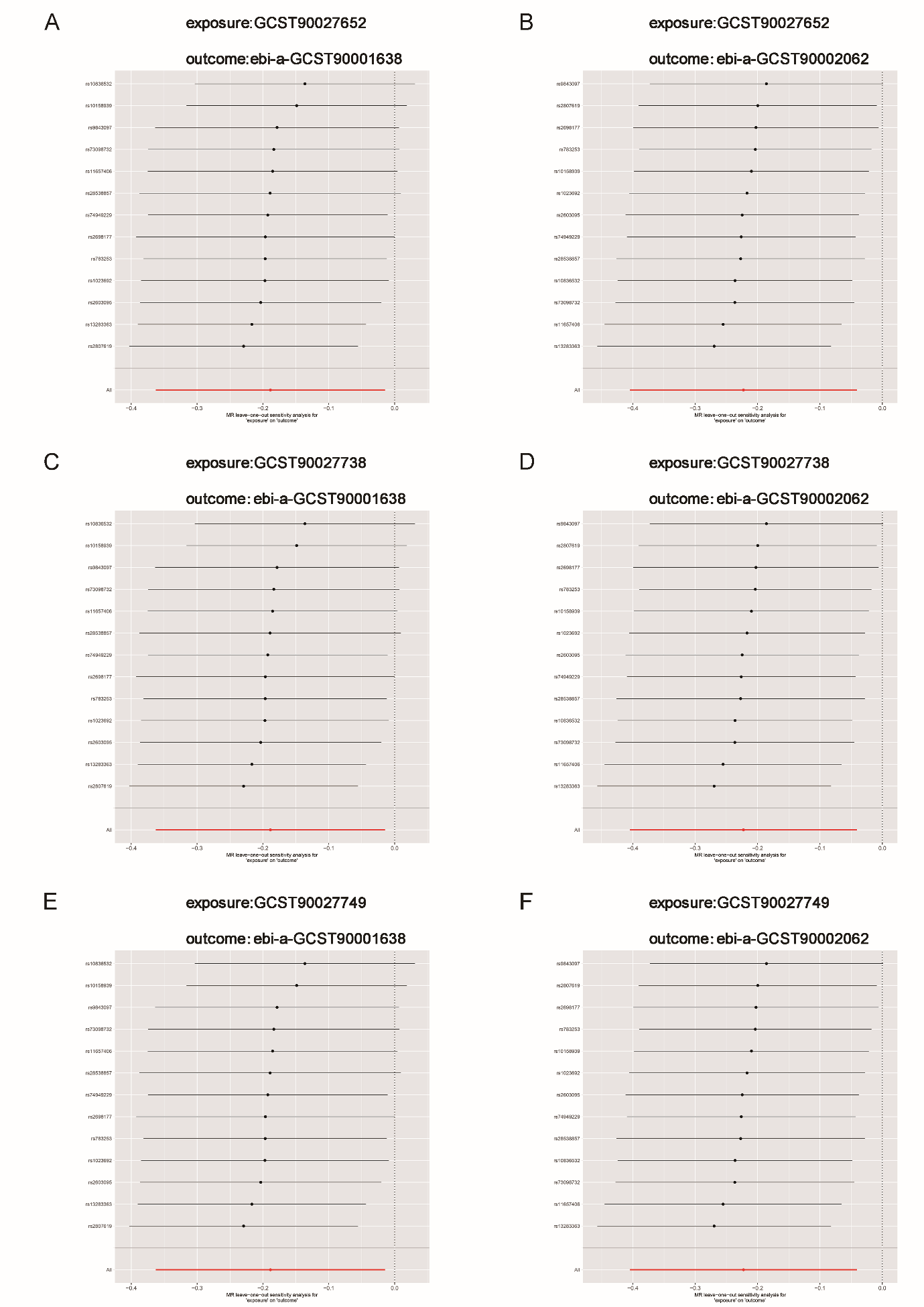
*

*Figure S9 Forest plot of the causal effect of the six different immunophenotypes on VTE risk via MR methods*

*
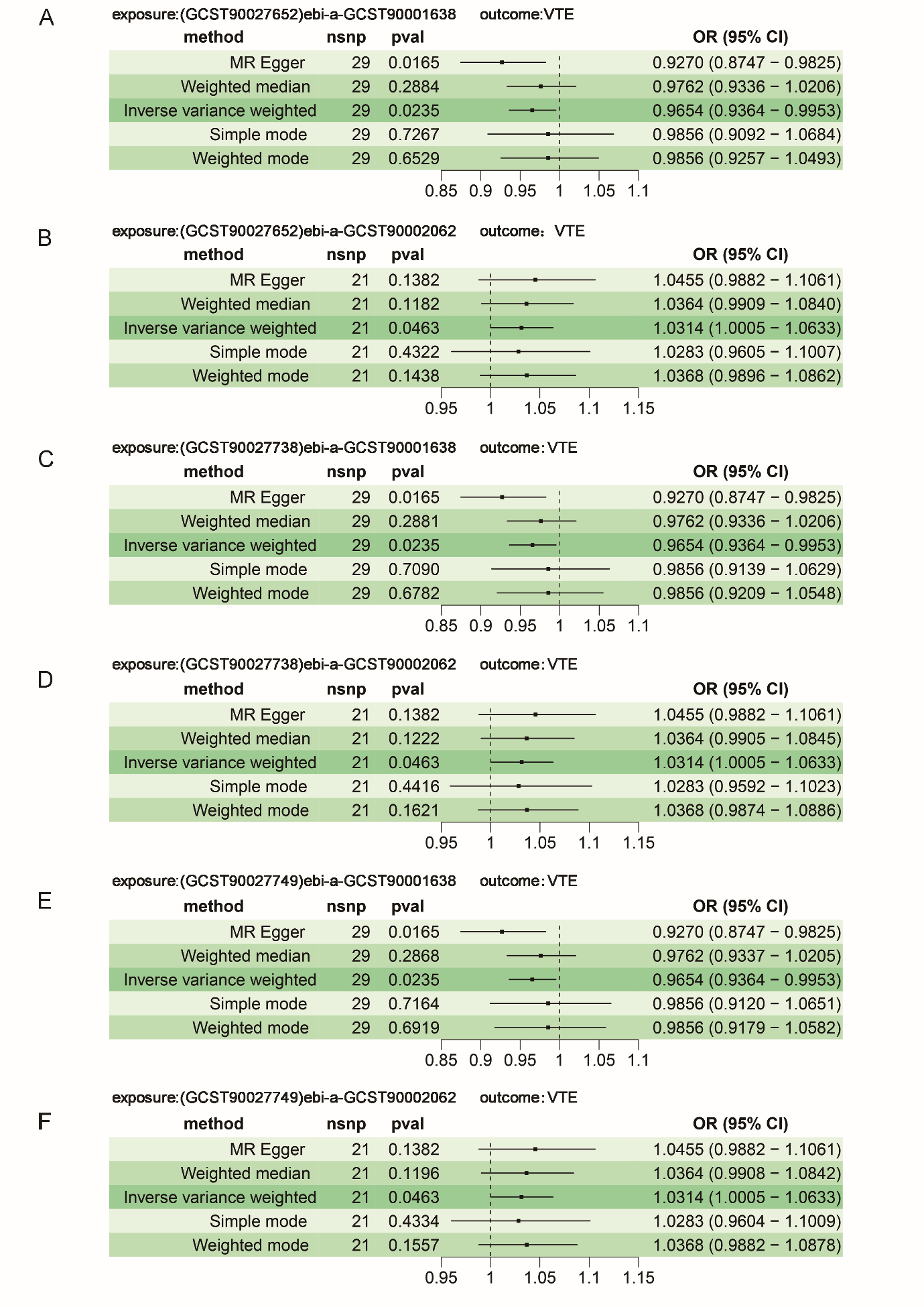
*

*Figure S10 Scatter plots for the causal association between the six different immunophenotypes and VTE*

*
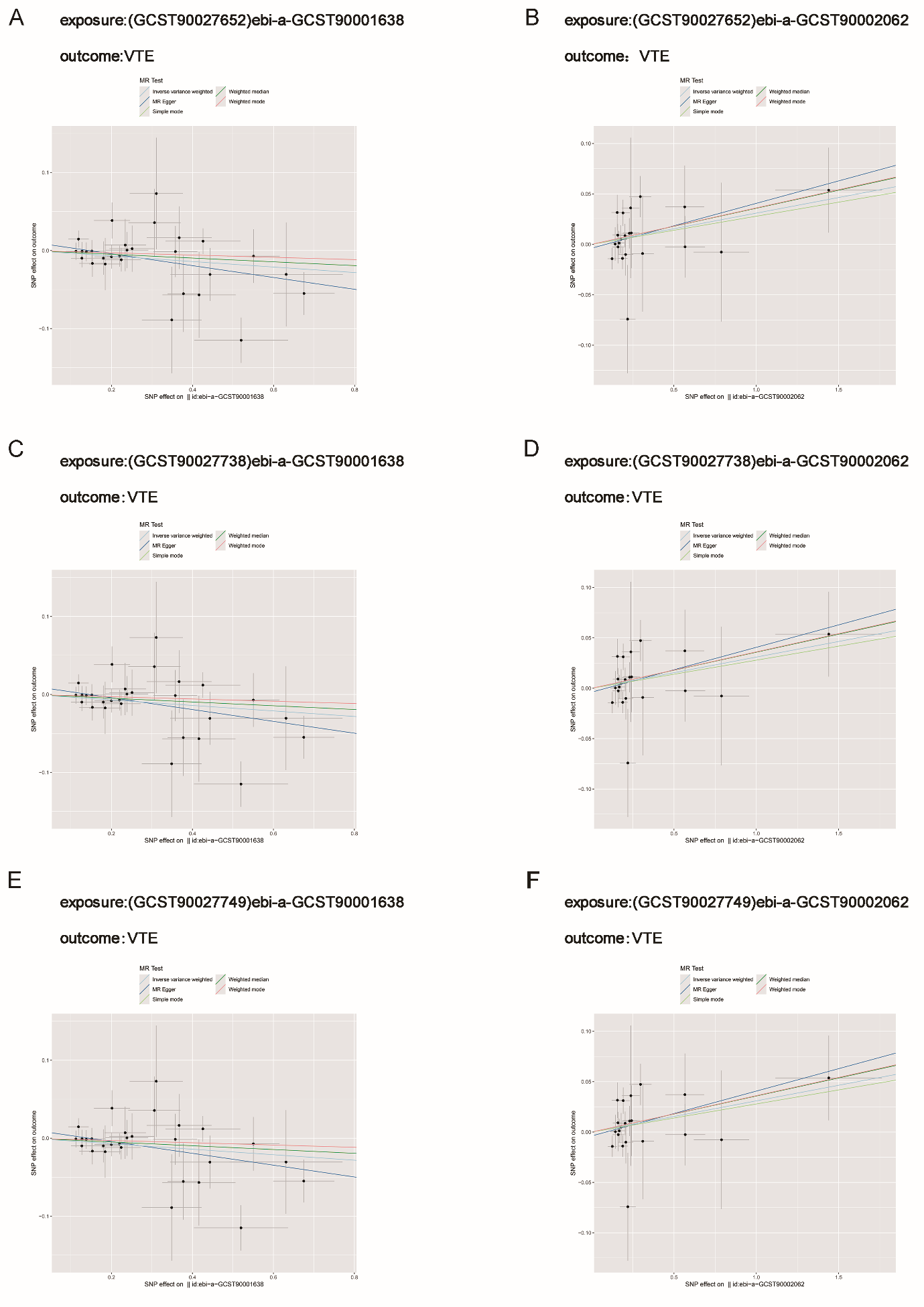
*

*Figure S11 Forest plot of the causal effect of the six different immunophenotypes on VTE*

*
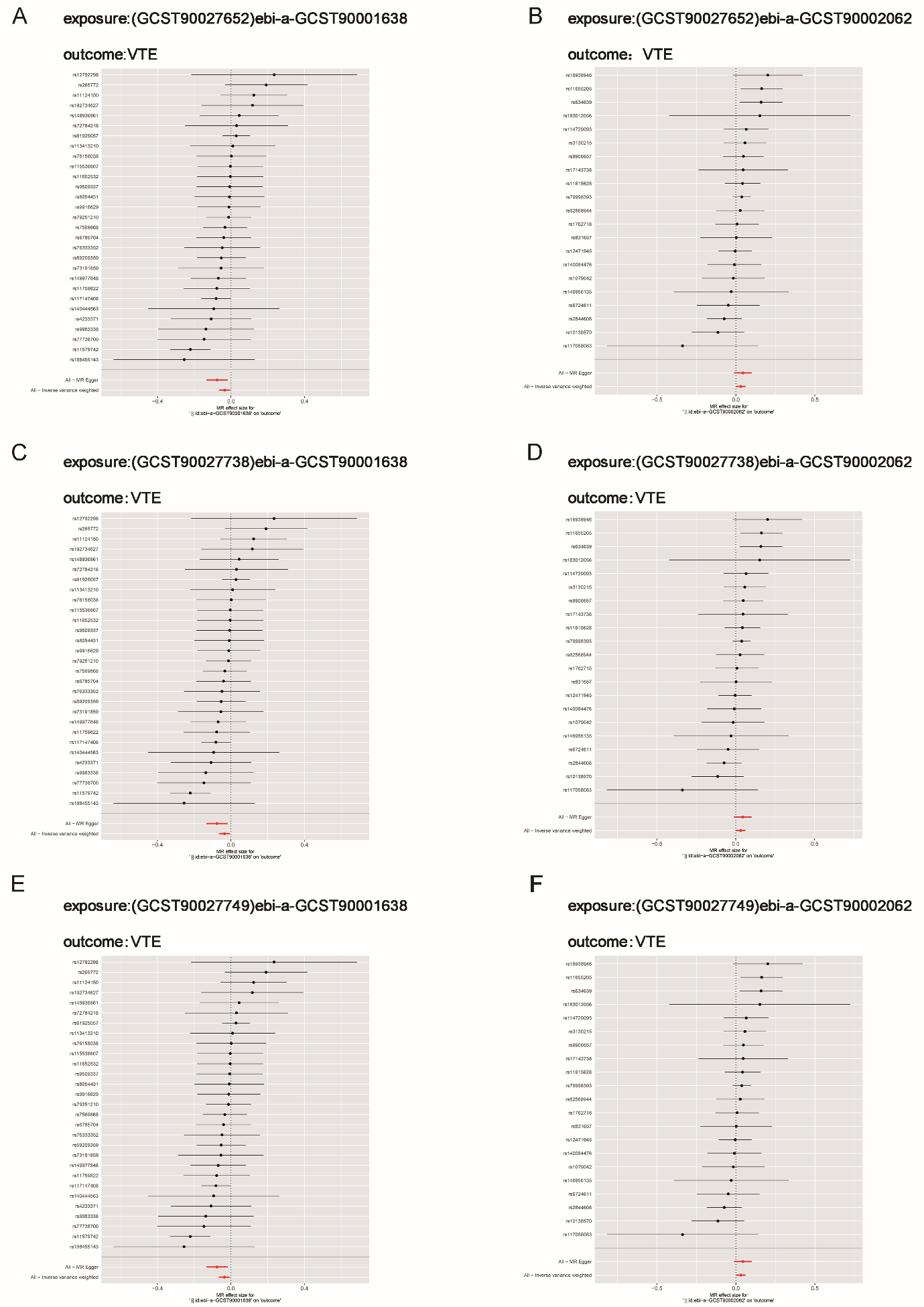
*

*Figure S12 Funnel plot of the causal effect of the six different immunophenotypes on VTE*

*
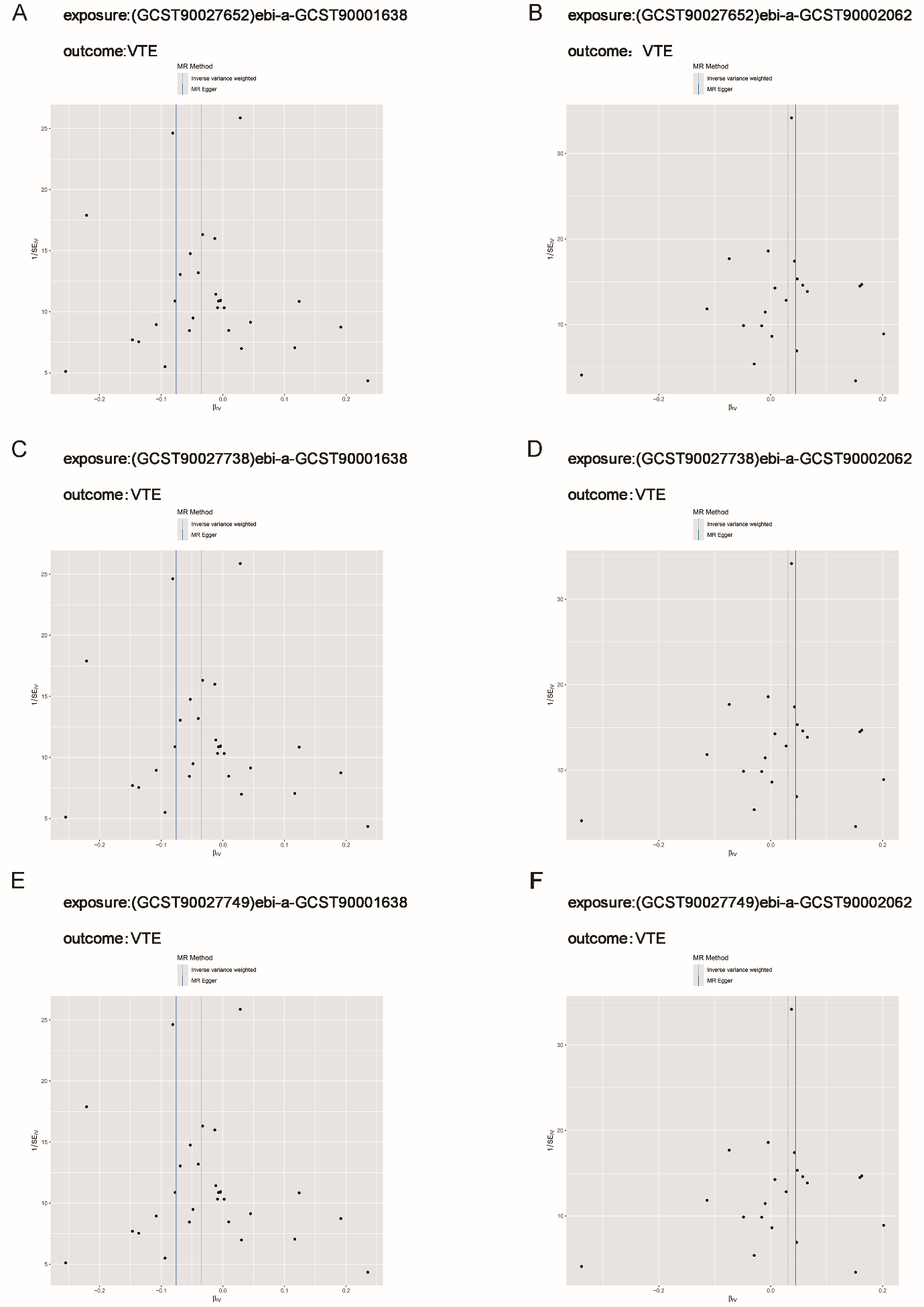
*

*Figure S13 Leave-one-out plots for the causal association between the six different immunophenotypes on VTE*

*
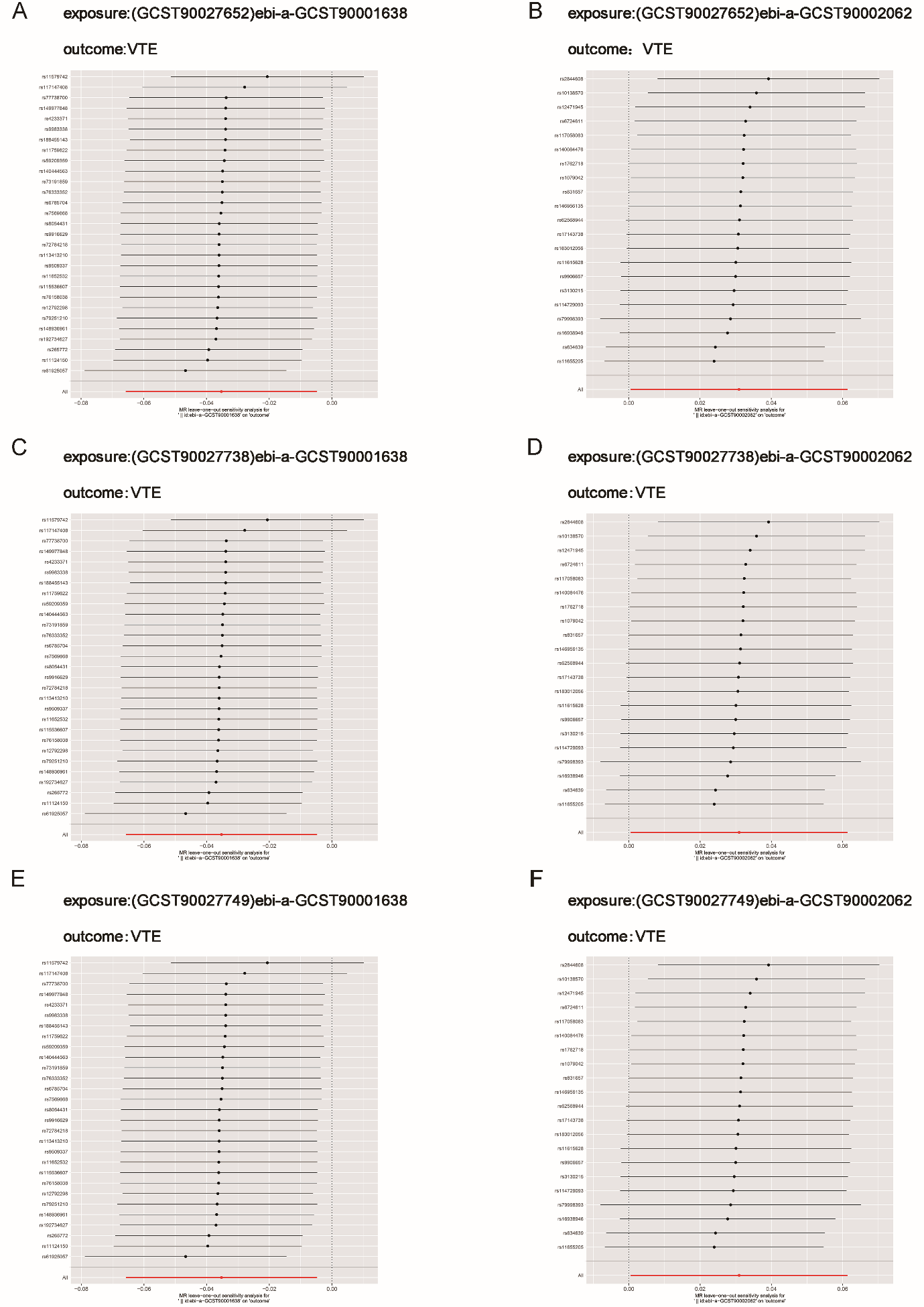
*
